# Supplementary material for: Tead1a Initiates Transcriptional Priming Through the TEAD1a/YAP‐Notch1‐Spi1/Cebpα Axis to Promote Neutrophil Fate
Source: Adv Sci (Weinh). 2025 Aug 28;12(41):e05441. doi: 10.1002/advs.202505441 (PMC12591127; doi:10.1002/advs.202505441)
Supplement: Supplementary file 4 — Supplementary Dataset [file ADVS-12-e05441-s004.zip › 3-5.zip!/3-5/02-5.cluster/cluster12/Enrich/ego_BP.pdf]

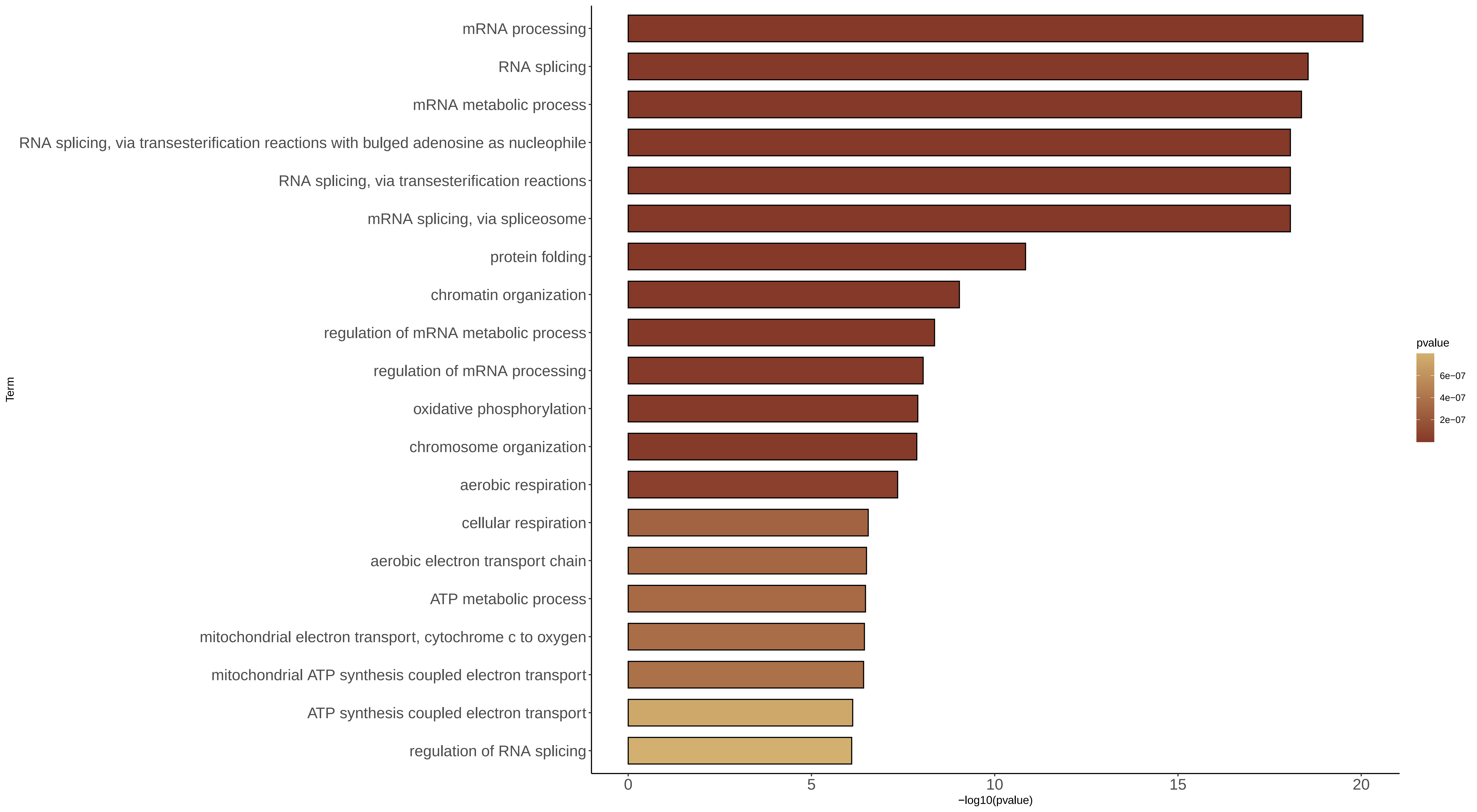
mRNA processing
RNA splicing
mRNA metabolic process
RNA splicing, via transesterification reactions with bulged adenosine as nucleophile
RNA splicing, via transesterification reactions
mRNA splicing, via spliceosome
protein folding
chromatin organization
regulation of mRNA metabolic process
regulation of mRNA processing
oxidative phosphorylation
chromosome organization
aerobic respiration
cellular respiration
aerobic electron transport chain
ATP metabolic process
mitochondrial electron transport, cytochrome c to oxygen
mitochondrial ATP synthesis coupled electron transport
ATP synthesis coupled electron transport
regulation of RNA splicing
Term
0
5
10
15
20
−log10(pvalue)
pvalue
6e−07
4e−07
2e−07
